# Supplementary material for: Species Diversity and Phylogenetic Relationships of Olive Lace Bugs (Hemiptera: Tingidae) Found in South Africa
Source: Insects. 2021 Sep 15;12(9):830. doi: 10.3390/insects12090830 (PMC8466438; doi:10.3390/insects12090830)
Supplement: Supplementary file 1 [file insects-12-00830-s001.zip › insects-1355462-supplementary.pdf]

Species diversity and phylogenetic relationships of olive lace bugs (Hemiptera: Tingidae) found in South Africa

Vaylen Hlaka, Éric Guilbert, Simon van Noort, Samuel J Smit, Elleunorah Allsopp, Jethro Langley and Barbara van Asch

SUPPLEMENTARY MATERIAL

Table S1. List of adult specimens of the olive lace bugs *Cysteochila lineata*, *Neoplerochila paliatseasi, Neoplerochila* sp. and *Plerochila australis* (Hemiptera: Tingidae) found in South Africa and used for imaging, DNA barcoding and sequencing of complete mitochondrial genomes. Cultivated host: *Olea europaea* subsp. *europaea*; Wild host*: Olea europaea* subsp. *cuspidata.* Hap – haplotype in neighbour-joining network for each species.

Specimen	Species	Collection date	Type of tree	Region	GPS coordinates	Use in this study	Hap	Genbank/SAM
P061	*Cysteochila lineata*	04-Feb-20	Wild ornamental	Stellenbosch	33°55'34.0"S 18°51'53.0"E	DNA barcode	Hap 1	MZ673445
P202	*Cysteochila lineata*	20-Feb-20	Wild ornamental	Stellenbosch	33°97'33.0"S 18°82'84.0"E	DNA barcode	Hap 14	MZ673446
P207	*Cysteochila lineata*	20-Feb-20	Wild ornamental	Stellenbosch	33°55'34.0"S 18°51'53.0"E	DNA barcode	Hap 5	MZ673447
P211	*Cysteochila lineata*	20-Feb-20	Wild ornamental	Stellenbosch	33°55'34.0"S 18°51'53.0"E	DNA barcode	Hap 7	MZ673448
P218	*Cysteochila lineata*	20-Feb-20	Wild ornamental	Stellenbosch	33°55'34.0"S 18°51'53.0"E	DNA barcode	Hap 6	MZ673449
P220	*Cysteochila lineata*	20-Feb-20	Wild ornamental	Stellenbosch	33°55'34.0"S 18°51'53.0"E	DNA barcode	Hap 11	MZ673450
P227	*Cysteochila lineata*	20-Feb-20	Wild ornamental	Stellenbosch	33°55'34.0"S 18°51'53.0"E	DNA barcode	Hap 15	MZ673451

P228	*Cysteochila lineata*	20-Feb-20	Wild ornamental	Stellenbosch	33°55'34.0"S 18°51'53.0"E	DNA barcode	Hap 5	MZ673452
P235	*Cysteochila lineata*	13-Mar-20	Cultivated on olive farm	Franschhoek	33°52'35.0"S 19°00'12.0"E	DNA barcode	Hap 5	MZ673453
P236	*Cysteochila lineata*	13-Mar-20	Cultivated on olive farm	Franschhoek	33°52'35.0"S 19°00'12.0"E	DNA barcode	Hap 13	MZ673454
P237	*Cysteochila lineata*	13-Mar-20	Cultivated on olive farm	Franschhoek	33°52'35.0"S 19°00'12.0"E	DNA barcode	Hap 18	MZ673455
P241	*Cysteochila lineata*	13-Mar-20	Cultivated on olive farm	Franschhoek	33°52'35.0"S 19°00'12.0"E	DNA barcode	Hap 5	MZ673456
P331	*Cysteochila lineata*	25-Nov-20	Wild on olive farm	Somerset West	34°02'31.0"S 18°45'20.0"E	DNA barcode	Hap 3	MZ673457
P332	*Cysteochila lineata*	25-Nov-20	Wild on olive farm	Somerset West	34°02'31.0"S 18°45'20.0"E	DNA barcode	Hap 3	MZ673458
P333	*Cysteochila lineata*	25-Nov-20	Wild on olive farm	Somerset West	34°02'31.0"S 18°45'20.0"E	DNA barcode	Hap 12	MZ673459
P334	*Cysteochila lineata*	25-Nov-20	Wild on olive farm	Somerset West	34°02'31.0"S 18°45'20.0"E	DNA barcode	Hap 4	MZ673460
P335	*Cysteochila lineata*	25-Nov-20	Wild on olive farm	Somerset West	34°02'31.0"S 18°45'20.0"E	DNA barcode	Hap 7	MZ673461
P338	*Cysteochila lineata*	25-Nov-20	Wild on olive farm	Somerset West	34°02'31.0"S 18°45'20.0"E	DNA barcode	Hap 17	MZ673462
P339	*Cysteochila lineata*	25-Nov-20	Wild on olive farm	Somerset West	34°02'31.0"S 18°45'20.0"E	DNA barcode	Hap 9	MZ673463
P340	*Cysteochila lineata*	25-Nov-20	Wild on olive farm	Somerset West	34°02'31.0"S 18°45'20.0"E	DNA barcode	Hap 8	MZ673464
P341	*Cysteochila lineata*	25-Nov-20	Wild on olive farm	Somerset West	34°02'31.0"S 18°45'20.0"E	DNA barcode	Hap 2	MZ673465
P342	*Cysteochila lineata*	26-Nov-20	Wild on olive farm	Somerset West	34°02'31.0"S 18°45'20.0"E	DNA barcode	Hap 16	MZ673466
P343	*Cysteochila lineata*	27-Nov-20	Wild on olive farm	Somerset West	34°02'31.0"S 18°45'20.0"E	DNA barcode	Hap 10	MZ673467
P344	*Cysteochila lineata*	28-Nov-20	Wild on olive farm	Somerset West	34°02'31.0"S 18°45'20.0"E	DNA barcode	Hap 7	MZ673468
P210	*Cysteochila lineata*	20-Feb-20	Wild ornamental	Stellenbosch	33°55'34.0"S 18°51'53.0"E	Imaging and deposit	n.a.	SAM-HEM-A012751
P238	*Cysteochila lineata*	13-Mar-20	Cultivated on olive farm	Franschhoek	33°52'35.0"S 19°00'12.0"E	Mitogenome	Hap 2	MZ935684
Np01	*Neoplerochila paliatseasi*	19-Mar-18	Cultivated ornamental	Brackenfell	33°56'27.0"S 18°42'01.3"E	DNA barcode (public data)	Hap 1	MN794060

Np21	*Neoplerochila paliatseasi*	19-Mar-18	Cultivated ornamental	Brackenfell	33°56'27.0"S 18°42'01.3"E	DNA barcode (public data)	Hap 1	MN794061
P002	*Neoplerochila paliatseasi*	11-Nov-15	Cultivated ornamental	Stellenbosch	33°56'13.0"S 18°49'11.4"E	DNA barcode (public data)	Hap 2	MN794062
P003	*Neoplerochila paliatseasi*	11-Nov-15	Cultivated ornamental	Stellenbosch	33°56'13.0"S 18°49'11.4"E	DNA barcode (public data)	Hap 2	MN794063
P006	*Neoplerochila paliatseasi*	11-Nov-15	Cultivated ornamental	Stellenbosch	33°56'13.0"S 18°49'11.4"E	DNA barcode (public data)	Hap 2	MN794064
P329	*Neoplerochila paliatseasi*	20-Nov-20	Wild ornamental	Pretoria	25°46'33.0"S 28°15'53.0"E	DNA barcode	Hap 1	MZ666853
P398	*Neoplerochila paliatseasi*	04-Feb-21	Cultivated on olive farm	Paarl	33°42'51.0"S 19°01'51.0"E	DNA barcode	Hap 4	MZ666854
P399	*Neoplerochila paliatseasi*	04-Feb-21	Cultivated on olive farm	Paarl	33°42'51.0"S 19°01'51.0"E	DNA barcode	Hap 4	MZ666855
P415	*Neoplerochila paliatseasi*	03-Mar-21	Cultivated on olive farm	Paarl	33°42'51.0"S 19°01'51.0"E	DNA barcode	Hap 1	MZ666856
P418	*Neoplerochila paliatseasi*	03-Mar-21	Wild on olive farm	Paarl	33°42'51.0"S 19°01'51.0"E	DNA barcode	Hap 5	MZ666857
P419	*Neoplerochila paliatseasi*	03-Mar-21	Wild on olive farm	Paarl	33°42'51.0"S 19°01'51.0"E	DNA barcode	Hap 5	MZ666858
P421	*Neoplerochila paliatseasi*	03-Mar-21	Wild on olive farm	Paarl	33°42'51.0"S 19°01'51.0"E	DNA barcode	Hap 1	MZ666859
P422	*Neoplerochila paliatseasi*	03-Mar-21	Wild on olive farm	Paarl	33°42'51.0"S 19°01'51.0"E	DNA barcode	Hap 5	MZ666860
P423	*Neoplerochila paliatseasi*	03-Mar-21	Wild on olive farm	Paarl	33°42'51.0"S 19°01'51.0"E	DNA barcode	Hap 5	MZ666861
P424	*Neoplerochila paliatseasi*	03-Mar-21	Wild on olive farm	Paarl	33°42'51.0"S 19°01'51.0"E	DNA barcode	Hap 3	MZ666862
P429	*Neoplerochila paliatseasi*	06-Mar-21	Cultivated on olive farm	Paarl	33°42'51.0"S 19°01'51.0"E	DNA barcode	Hap 5	MZ666863
Np02	*Neoplerochila paliatseasi*	19-Mar-18	Cultivated ornamental	Brackenfell	33°56'27.0"S 18°42'01.3"E	Mitogenome (public data)	Hap 1	NC_046031
Np06	*Neoplerochila paliatseasi*	19-Mar-18	Cultivated ornamental	Brackenfell	33°53'12.0"S 18°41'53.0"E	Imaging and deposit	n.a.	SAM-HEM-A011647
P396	*Neoplerochila* sp.	04-Feb-21	Cultivated on olive farm	Paarl	33°42'51.0"S 19°01'51.0"E	DNA barcode	n.a.	MZ673417
P400	*Neoplerochila* sp.	04-Feb-21	Cultivated on olive farm	Paarl	33°42'51.0"S 19°01'51.0"E	DNA barcode	n.a.	MZ673418
P414	*Neoplerochila* sp.	03-Mar-21	Cultivated on olive farm	Paarl	33°42'51.0"S 19°01'51.0"E	DNA barcode	n.a.	MZ673419

P416	*Neoplerochila* sp.	03-Mar-21	Cultivated on olive farm	Paarl	33°42'51.0"S 19°01'51.0"E	DNA barcode	n.a.	MZ673420
P417	*Neoplerochila* sp.	03-Mar-21	Wild on olive farm	Paarl	33°42'51.0"S 19°01'51.0"E	DNA barcode	n.a.	MZ673421
P425	*Neoplerochila* sp.	06-Mar-21	Cultivated on olive farm	Paarl	33°42'51.0"S 19°01'51.0"E	DNA barcode	n.a.	MZ673422
P426	*Neoplerochila* sp.	06-Mar-21	Cultivated on olive farm	Paarl	33°42'51.0"S 19°01'51.0"E	DNA barcode	n.a.	MZ673423
P428	*Neoplerochila* sp.	06-Mar-21	Cultivated on olive farm	Paarl	33°42'51.0"S 19°01'51.0"E	DNA barcode	n.a.	MZ673424
P430	*Neoplerochila* sp.	06-Mar-21	Cultivated on olive farm	Paarl	33°42'51.0"S 19°01'51.0"E	DNA barcode	n.a.	MZ673425
P431	*Neoplerochila* sp.	06-Mar-21	Cultivated on olive farm	Paarl	33°42'51.0"S 19°01'51.0"E	DNA barcode	n.a.	MZ673426
P432	*Neoplerochila* sp.	06-Mar-21	Cultivated on olive farm	Paarl	33°42'51.0"S 19°01'51.0"E	DNA barcode	n.a.	MZ673427
P433	*Neoplerochila* sp.	06-Mar-21	Cultivated on olive farm	Paarl	33°42'51.0"S 19°01'51.0"E	DNA barcode	n.a.	MZ673428
P434	*Neoplerochila* sp.	06-Mar-21	Cultivated on olive farm	Paarl	33°42'51.0"S 19°01'51.0"E	DNA barcode	n.a.	MZ673429
P405	*Neoplerochila* sp.	04-Feb-21	Cultivated on olive farm	Paarl	33°42'51.0"S 19°01'51.0"E	Imaging and deposit	n.a.	SAM-HEM-A012753
P401	*Neoplerochila* sp.	05-Feb-21	Cultivated on olive farm	Paarl	33°42'51.0"S 19°01'51.0"E	Mitogenome	n.a.	MZ935686
P025	*Plerochila australis*	08-Nov-15	Cultivated ornamental	Stellenbosch	33°54'10.6"S 18°48'46.1"E	DNA barcode	Hap 1	MZ676957
P026	*Plerochila australis*	08-Nov-15	Cultivated ornamental	Stellenbosch	33°54'10.6"S 18°48'46.1"E	DNA barcode	Hap 3	MZ676958
P031	*Plerochila australis*	08-Nov-15	Cultivated ornamental	Stellenbosch	33°54'10.6"S 18°48'46.1"E	DNA barcode	Hap 2	MZ676959
P032	*Plerochila australis*	30-Dec-15	Cultivated ornamental	Stellenbosch	33°56'13.2"S 18°51'13.2"E	DNA barcode	Hap 2	MZ676960
P035	*Plerochila australis*	04-Mar-16	Wild ornamental	Stanford	34°26'19.2"S 19°27'06.9"E	DNA barcode	Hap 4	MZ676961
P052	*Plerochila australis*	16-Oct-16	Cultivated ornamental	Stellenbosch	33°58'07.2"S 18°55'56.8"E	DNA barcode	Hap 5	MZ676962
P062	*Plerochila australis*	04-Feb-20	Wild ornamental	Stellenbosch	33°55'34.0"S 18°51'53.0"E	DNA barcode	Hap 10	MZ676963
P064	*Plerochila australis*	26-Nov-19	Cultivated ornamental	Stellenbosch	33°97'33.0"S 18°82'84.0"E	DNA barcode	Hap 6	MZ676964

P065	*Plerochila australis*	04-Feb-20	Cultivated ornamental	Stellenbosch	33°97'33.0"S 18°82'84.0"E	DNA barcode	Hap 12	MZ676965
P066	*Plerochila australis*	26-Nov-19	Cultivated ornamental	Stellenbosch	33°97'33.0"S 18°82'84.0"E	DNA barcode	Hap 10	MZ676966
P067	*Plerochila australis*	26-Nov-19	Cultivated ornamental	Stellenbosch	33°97'33.0"S 18°82'84.0"E	DNA barcode	Hap 8	MZ676967
P068	*Plerochila australis*	26-Nov-19	Cultivated ornamental	Stellenbosch	33°97'33.0"S 18°82'84.0"E	DNA barcode	Hap 11	MZ676968
P069	*Plerochila australis*	26-Nov-19	Cultivated ornamental	Stellenbosch	33°97'33.0"S 18°82'84.0"E	DNA barcode	Hap 8	MZ676969
P070	*Plerochila australis*	26-Nov-19	Cultivated ornamental	Stellenbosch	33°97'33.0"S 18°82'84.0"E	DNA barcode	Hap 6	MZ676970
P071	*Plerochila australis*	26-Nov-19	Cultivated ornamental	Stellenbosch	33°97'33.0"S 18°82'84.0"E	DNA barcode	Hap 11	MZ676971
P072	*Plerochila australis*	26-Nov-19	Cultivated ornamental	Stellenbosch	33°97'33.0"S 18°82'84.0"E	DNA barcode	Hap 8	MZ676972
P073	*Plerochila australis*	26-Nov-19	Cultivated ornamental	Stellenbosch	33°97'33.0"S 18°82'84.0"E	DNA barcode	Hap 11	MZ676973
P074	*Plerochila australis*	26-Nov-19	Cultivated ornamental	Stellenbosch	33°97'33.0"S 18°82'84.0"E	DNA barcode	Hap 7	MZ676974
P075	*Plerochila australis*	26-Nov-19	Cultivated ornamental	Stellenbosch	33°97'33.0"S 18°82'84.0"E	DNA barcode	Hap 11	MZ676975
P076	*Plerochila australis*	26-Nov-19	Cultivated ornamental	Stellenbosch	33°97'33.0"S 18°82'84.0"E	DNA barcode	Hap 9	MZ676976
P077	*Plerochila australis*	26-Nov-19	Cultivated ornamental	Stellenbosch	33°97'33.0"S 18°82'84.0"E	DNA barcode	Hap 6	MZ676977
P081	*Plerochila australis*	26-Nov-19	Cultivated ornamental	Stellenbosch	33°97'33.0"S 18°82'84.0"E	DNA barcode	Hap 6	MZ676978
P083	*Plerochila australis*	26-Nov-19	Cultivated ornamental	Stellenbosch	33°97'33.0"S 18°82'84.0"E	DNA barcode	Hap 12	MZ676979
P087	*Plerochila australis*	26-Nov-19	Cultivated ornamental	Stellenbosch	33°97'33.0"S 18°82'84.0"E	DNA barcode	Hap 11	MZ676980
P088	*Plerochila australis*	26-Nov-19	Cultivated ornamental	Stellenbosch	33°97'33.0"S 18°82'84.0"E	DNA barcode	Hap 12	MZ676981
P089	*Plerochila australis*	26-Nov-19	Cultivated ornamental	Stellenbosch	33°97'33.0"S 18°82'84.0"E	DNA barcode	Hap 11	MZ676982
P320	*Plerochila australis*	20-Nov-20	Wild ornamental	Pretoria	25°46'33.0"S 28°15'53.0"E	DNA barcode	Hap 14	MZ676983
P321	*Plerochila australis*	20-Nov-20	Wild ornamental	Pretoria	25°46'33.0"S 28°15'53.0"E	DNA barcode	Hap 13	MZ676983

P322	*Plerochila australis*	20-Nov-20	Wild ornamental	Pretoria	25°46'33.0"S 28°15'53.0"E	DNA barcode	Hap 12	MZ676985
P323	*Plerochila australis*	20-Nov-20	Wild ornamental	Pretoria	25°46'33.0"S 28°15'53.0"E	DNA barcode	Hap 11	MZ676986
P324	*Plerochila australis*	20-Nov-20	Wild ornamental	Pretoria	25°46'33.0"S 28°15'53.0"E	DNA barcode	Hap 11	MZ676987
P030	*Plerochila australis*	08-Nov-15	Cultivated ornamental	Stellenbosch	33°54'11.0"S 18°48'54.0"E	Imaging and deposit	n.a.	SAM-HEM-A010383
P028	*Plerochila australis*	08-Nov-15	Cultivated ornamental	Stellenbosch	33°54'10.6"S 18°48'46.1"E	Mitogenome	Hap 12	MZ935685

Table S2. PCR primers used for amplification of the standard COI barcoding region (~700 bp) in the olive lace bugs *Cysteochila lineata*, *Neoplerochila paliatseasi*, *Neoplerochila* sp. and *Plerochila australis* (Hemiptera: Tingidae). *Primer used for unidirectional Sanger sequencing.

Species	Primer	Sequence (5' - 3')	Reference
Cysteochila lineata	Cys-F	CAACCAATCACAAAGATATCGG	This study
	Cys-R*	ACTTCAGGATGTCCGAAAAATC	
Plerochila australis	Ple-F	CAACTAACCACAAAGATATCGG	
	Ple-R*	TTCAGGGTGCCCGAAAAATCA	
Neoplerochila paliatseasi and *Neoplerochila* sp.	Neo-F	CGACTAATCACAAAGACATCGG	Langley et al. 2020
	Neo-R*	CTTCGGGATGTCCAAAGAATC	

Table S3. Mitochondrial genomes of Tingidae used to assess the phylogenetic position of the four olive lace bugs *Cysteochila lineata*, *Neoplerochila paliatseasi*, *Neoplerochila* sp. and *Plerochila australis* (Hemiptera: Tingidae) found in South Africa. *Miridae species used as outgroup.

Agramma huperhanum	NC_037146.1	Liu et al., 2018

Ammianus toi	JQ739178.1	Li et al., 2017
Corythucha ciliata	NC_022922.1	Yang et al., 2013
Corythucha marmorata	MG479390.1	Lin et al., 2017
Cysteochila chiniana	NC_037833.1	Yang et al., 2018
Cysteochila lineata	Upon acceptance	This study
Dictyla platyoma	NC_037834.1	Yang et al., 2018
Metasalis populi	NC_037835.1	Yang et al., 2018
Neoplerochila paliatseasi	MN794065	Langley et al., 2020
Neoplerochila sp.	Upon acceptance	This study
Perissonemia borneensis	KU896785.1	Liu et al., 2018
Phatnoma laciniatum	NC_037148.1	Liu et al., 2018
Plerochila australis	Upon acceptance	This study
Pseudacysta perseae	NC_025299.1	Kocher et al., 2015
Stephanitis chinensis	MF498769.1	Li et al., 2017
Stephanitis mendica	JQ739184.1	Li et al., 2017
Tingis cardui	NC_037836	Yang et al., 2018
Trachypeplus jacobsoni	NC_037837.1	Yang et al., 2018
*Adelphocoris fasciaticollis**	NC_023796.1	Wang et al., 2016
*Apolygus lucorum**	NC_023083.1	Wang et al., 2014

Table S4. Intraspecific p-distances (%) in 30 species in the family Tingidae (Hemiptera), based on a 501 bp alignment of COI barcoding sequences (n = 349).

**Species**	**n**	**Max**	**Min**	**Mean**	**SE**
Acalypta elegans	36	1.42	0.00	0.48	0.20
Acalypta musci	4	0.40	0.00	0.27	0.18
Acalypta nigrina	3	0.00	0.00	0.00	0.00
Acalypta parvula	12	0.60	0.00	0.20	0.12

Catoplatus fabricii	4	0.00	0.00	0.00	0.00
Copium clavicorne	4	0.00	0.00	0.00	0.00
Corythucha ciliata	20	1.21	0.00	0.52	0.22
Corythucha immaculata	3	0.60	0.00	0.40	0.23
Corythucha juglandis	3	2.24	0.00	1.50	0.46
Corythucha marmorata	53	2.66	0.00	0.63	0.15
Corythucha pallipes	3	0.20	0.00	0.13	0.13
Cysteochila lineata	25	1.83	0.00	0.78	0.19
Derephysia foliacea	18	9.28	0.00	1.04	0.16
Dictyla humuli	5	0.60	0.00	0.32	0.19
Dictyonota strichnocera	4	0.40	0.00	0.20	0.14
Gargaphia opacula	5	0.40	0.00	0.24	0.14
Gargaphia tiliae	12	0.40	0.00	0.20	0.13
Hesperotingis fuscata	4	0.60	0.00	0.47	0.22
Kalama tricornis	10	0.40	0.00	0.08	0.05
Lasiacantha capucina	6	0.60	0.00	0.28	0.16
Neoplerochila paliatseasi	17	1.41	0.00	0.52	0.21
Neoplerochila sp.	14	0.00	0.00	0.00	0.00
Oncochila simplex	7	0.40	0.00	0.15	0.11
Physatocheila variegata	37	1.01	0.00	0.22	0.10
Plerochila australis	31	3.29	0.00	1.49	0.35
Stephanitis takeyai	4	0.20	0.00	0.10	0.10
Stephanitis typica	3	1.61	0.00	1.08	0.37
Tingis cardui	11	1.83	0.00	1.07	0.31
Tingis crispata	6	2.44	0.00	1.27	0.32
Tingis reticulata	3	0.00	0.00	0.00	0.00

Table S5. Interspecific mean p-distances (%) among 30 species in the family Tingidae (Hemiptera), based on a 501 bp alignment of COI barcoding sequences (n = 349).

	**Species**	**1**	**2**	**3**	**4**	**5**	**6**	**7**	**8**	**9**	**10**	**11**	**12**	**13**	**14**	**15**	**16**	**17**	**18**	**19**	**20**	**21**	**22**	**23**	**24**	**25**	**26**	**27**	**28**	**29**	**30**
**1**	*Acalypta elegans*		2.25	1.91	2.20	2.26	2.21	2.33	2.20	2.24	2.32	2.40	2.66	2.22	2.16	2.27	2.22	2.17	2.45	2.38	2.15	2.82	2.96	2.21	2.26	2.58	2.07	2.34	2.40	2.49	2.20
**2**	*Acalypta musci*	19.03		2.50	2.43	2.45	2.43	2.54	2.48	2.46	2.40	2.61	2.45	2.11	2.70	2.37	2.49	2.60	2.61	2.31	2.47	2.42	2.48	2.50	2.38	2.48	2.27	2.42	2.44	2.50	2.43
**3**	*Acalypta nigrina*	14.50	22.83		2.31	2.35	2.08	2.21	2.22	2.23	2.37	2.44	2.80	2.21	2.38	2.44	2.18	2.09	2.20	2.21	2.35	2.46	2.58	2.25	2.38	2.34	2.08	2.26	2.27	2.45	2.40
**4**	*Acalypta parvula*	19.44	22.12	20.48		2.39	2.13	2.38	2.26	2.32	2.32	2.38	2.80	2.47	2.20	2.07	2.25	2.45	2.32	2.22	2.31	2.46	2.47	2.28	2.29	2.59	2.26	2.18	2.39	2.52	2.27
**5**	*Catoplatus fabricii*	20.03	22.76	21.49	21.28		2.18	2.23	2.16	2.21	2.31	2.22	2.44	2.36	2.33	2.16	2.26	2.11	2.26	2.22	2.32	2.40	2.49	2.33	2.21	2.23	2.24	2.11	2.27	2.14	2.15
**6**	*Copium clavicorne*	19.07	21.52	17.31	18.26	18.10		2.20	2.08	2.18	2.05	2.16	2.51	1.89	2.00	2.04	1.79	2.18	2.09	1.86	2.18	2.36	2.45	2.07	1.87	2.36	2.00	2.12	2.06	2.28	1.99
**7**	*Corythucha ciliata*	21.32	23.06	19.70	21.86	19.18	18.99		1.58	0.99	2.04	1.44	2.54	2.25	2.22	2.26	2.27	2.02	2.20	2.25	2.36	2.08	2.21	1.95	2.29	2.57	2.00	2.14	1.95	2.44	2.11
**8**	*Corythucha immaculata*	19.06	24.00	18.85	21.21	18.83	17.31	11.61		1.55	1.89	1.70	2.61	2.21	2.23	2.21	2.20	2.07	2.14	2.20	2.22	2.16	2.39	2.20	2.21	2.30	1.97	2.15	2.07	2.29	2.18
**9**	*Corythucha juglandis*	20.63	22.89	19.79	21.39	19.44	18.83	5.22	11.03		2.03	1.33	2.50	2.21	2.18	2.26	2.17	1.94	2.28	2.16	2.19	2.03	2.19	2.03	2.20	2.46	1.87	2.08	2.04	2.27	2.06
**10**	*Corythucha marmorata*	19.97	22.34	20.51	21.23	20.67	16.59	16.94	15.35	17.03		2.02	2.61	2.21	2.07	2.16	2.19	2.13	2.19	2.18	2.12	2.35	2.48	1.89	1.98	2.39	1.76	2.04	2.08	2.28	1.94
**11**	*Corythucha pallipes*	21.64	24.39	21.81	22.81	19.93	17.99	8.94	12.93	8.49	16.17		2.49	2.30	2.12	2.31	2.13	2.01	2.42	2.44	2.32	2.31	2.38	1.95	2.36	2.45	1.93	2.16	2.12	2.40	2.07

**12**	*Cysteochila lineata*	25.07	22.69	26.75	27.23	23.36	23.17	23.58	24.13	22.55	24.68	22.68		2.43	2.51	2.75	2.57	2.77	2.69	2.78	2.48	2.27	2.28	2.68	2.55	1.97	2.62	2.64	2.54	2.49	2.51
**13**	*Derephysia foliacea*	19.39	18.80	18.93	22.52	21.20	14.99	20.00	19.49	19.83	19.75	21.25	23.10		2.00	2.31	2.06	2.28	2.32	2.12	2.00	2.49	2.59	2.11	1.93	2.22	2.17	2.23	2.25	2.27	2.14
**14**	*Dictyla humuli*	20.16	26.27	21.29	19.47	20.48	17.27	20.40	19.41	19.67	17.90	19.03	23.06	18.29		2.26	2.17	2.13	2.28	2.28	2.24	2.33	2.51	2.13	2.08	2.36	2.03	2.25	2.17	2.15	1.94
**15**	*Dictyonota strichnocera*	19.50	21.62	21.10	17.98	18.79	17.14	19.79	19.86	20.43	19.71	20.18	25.71	20.80	20.11		2.23	2.37	2.32	2.01	2.22	2.40	2.60	2.32	2.25	2.54	1.94	2.17	2.40	2.44	2.09
**16**	*Gargaphia opacula*	18.52	22.78	18.54	19.51	18.66	13.80	19.37	18.42	18.52	19.05	17.84	23.86	17.45	18.86	18.34		1.90	2.00	2.04	2.09	2.42	2.62	2.27	2.28	2.42	2.01	2.15	2.18	2.28	2.06
**17**	*Gargaphia tiliae*	19.86	24.26	19.07	22.02	18.83	18.47	17.11	17.70	16.43	17.94	18.09	26.97	20.09	18.75	21.26	15.25		2.07	2.34	2.29	2.40	2.39	2.09	2.35	2.43	2.03	2.23	2.20	2.17	2.40
**18**	*Hesperotingis fuscata*	22.52	24.43	19.98	19.81	18.93	17.95	19.13	19.52	20.54	19.08	22.24	25.67	20.81	19.49	20.67	16.99	17.82		2.09	2.33	2.33	2.37	2.47	2.17	2.48	2.33	2.32	2.17	2.40	2.28
**19**	*Kalama tricornis*	21.22	20.86	18.72	20.43	19.36	15.02	19.88	19.44	19.17	18.48	22.61	25.52	18.49	20.73	17.90	17.27	21.57	18.66		2.37	2.42	2.49	2.32	2.07	2.39	2.03	2.09	2.06	2.45	2.03
**20**	*Lasiacantha capucina*	17.60	22.55	20.58	22.10	20.48	18.53	20.93	19.26	19.04	18.95	20.98	23.12	17.31	19.69	20.10	17.88	19.53	21.51	20.59		2.65	2.76	2.22	2.17	2.16	2.19	1.97	2.19	2.36	2.07
**21**	*Neoplerochila paliatseasi*	27.64	22.92	23.25	22.25	22.33	21.10	17.52	18.98	17.29	21.49	19.88	20.62	24.51	21.91	22.61	21.96	22.05	20.43	22.96	24.71		1.24	2.42	2.31	2.10	2.00	2.19	2.16	2.35	2.33
**22**	*Neoplerochila sp.*	28.72	23.82	24.67	22.59	23.95	21.72	19.08	22.19	19.14	22.97	21.29	20.15	25.41	24.44	25.38	24.30	22.63	20.40	23.24	25.98	6.96		2.62	2.53	2.32	2.23	2.45	2.27	2.59	2.42
**23**	*Oncochila simplex*	19.14	22.08	19.49	20.51	20.18	16.31	15.79	19.25	16.88	14.72	15.68	24.33	18.33	18.66	20.87	19.06	16.87	22.05	20.61	19.63	21.95	24.08		2.04	2.52	2.12	1.85	2.31	2.36	2.00
**24**	*Physatochiela variegata*	19.30	21.86	21.77	21.14	18.66	15.00	20.76	19.52	19.85	17.67	21.63	23.87	15.19	18.12	21.53	19.89	19.92	18.54	18.28	18.27	19.86	22.05	17.05		2.22	2.07	1.97	2.29	2.20	2.09

25	*Plerochila australis*	23.36	23.56	20.73	25.13	19.98	21.23	24.65	20.85	23.34	21.76	23.23	16.70	19.66	21.08	23.31	21.83	21.45	23.24	22.24	18.69	19.24	21.52	22.75	19.42		2.28	2.10	2.42	2.41	2.43
26	*Stephanitis takeyai*	17.12	20.58	17.04	20.82	20.13	16.07	16.65	16.26	15.48	13.85	15.75	23.04	19.17	17.16	15.68	17.02	17.90	21.26	17.30	19.25	17.44	20.09	18.02	18.56	20.00		1.78	1.92	2.26	1.80
27	*Stephanitis typica*	20.16	22.42	19.36	20.58	18.69	17.99	18.42	18.63	18.18	17.60	18.77	25.11	19.97	19.83	19.35	18.29	19.65	20.82	19.05	17.03	19.67	22.93	15.32	16.21	18.20	13.87		2.19	2.17	1.91
28	*Tingis cardui*	24.36	23.41	21.44	23.02	21.01	17.89	16.80	18.88	17.88	19.18	18.67	23.78	21.01	19.73	22.85	20.19	18.88	18.60	19.18	19.90	19.16	20.41	21.20	21.17	23.49	17.02	21.07		2.15	2.08
29	*Tingis crispata*	23.97	24.16	23.30	23.35	19.03	21.10	22.82	21.50	21.44	21.24	23.09	23.76	22.01	19.34	23.47	20.32	19.66	23.07	22.89	22.31	22.47	24.88	21.87	19.89	22.83	21.34	20.61	19.29		2.14
30	*Tingis reticulata*	18.69	22.79	20.94	20.77	17.31	16.53	18.34	18.90	18.29	15.52	18.51	22.19	19.24	15.36	18.61	17.25	21.63	20.28	17.49	17.86	21.11	22.49	16.58	17.76	22.06	14.57	15.26	18.46	20.17	
	Max	***28.72***	***26.27***	***26.75***	***27.23***	***23.95***	***23.17***	***24.65***	***24.13***	***23.34***	***24.68***	***23.23***	***26.97***	***25.41***	***24.44***	***25.38***	***24.30***	***22.63***	***23.24***	***23.24***	***25.98***	***22.47***	***24.88***	***22.75***	***21.17***	***23.49***	***21.34***	***21.07***	***19.29***	***20.17***	***28.72***
	Min	***14.50***	***18.80***	***17.04***	***17.98***	***17.31***	***13.80***	***5.22***	***11.03***	***8.49***	***13.85***	***15.68***	***16.70***	***15.19***	***15.36***	***15.68***	***15.25***	***16.87***	***18.54***	***17.30***	***17.03***	***6.96***	***20.09***	***15.32***	***16.21***	***18.20***	***13.87***	***15.26***	***18.46***	***20.17***	***5.22***

Table S6. Main features of the complete mitochondrial genomes of *Cysteochila lineata*, *Neoplerochila* sp. and *Plerochila australis* (Hemiptera: Tingidae). J – majority strand; N – minority strand; IGN – number of intergenic nucleotides (negative values indicate gene overlap).

				Cysteochila lineata					***Plerochila australis***					***Neoplerochila* sp.**				
**Gene/region**	**Code**	**Strand**	**Anticodon**	**Coordinates**	**Size (bp)**	**Start**	**Stop**	**IGN**	**Coordinates**	**Size (bp)**	**Start**	**Stop**	**IGN**	**Coordinates**	**Size (bp)**	**Start**	**Stop**	**IGN**
tRNA^{Ile}	I	J	GAT	1-63	63	-	-	-	1-64	64	-	-	-	1-64	64	-	-	-
tRNA^{Gln}	Q	N	TTG	61-128	68	-	-	-3	61-130	70	-	-	-4	61-128	68	-	-	-4

tRNA^{Met}	M	J	CAT	127-193	67	-	-	-2	141-204	64	-	-	10	127-193	67	-	-	-2
ND2	-	J	-	193-1,162	970	ATT	T__	-1	205-1,176	972	ATT	TAA	0	193-1,164	972	ATT	TAA	-1
tRNA^{Trp}	W	J	TCA	1,162-1,230	69	-	-	-1	1,174-1,241	68	-	-	-3	1,166-1,236	71	-	-	1
tRNA^{Cys}	C	N	GCA	1,222-1,287	66	-	-	-9	1,233-1,295	63	-	-	-9	1,226-1,288	63	-	-	-11
tRNA^{Tyr}	Y	N	GTA	1,297-1,360	64	-	-	9	1,298-1,361	64	-	-	2	1,301-1,364	64	-	-	12
COI	-	J	-	1,365-2,900	1,536	ATG	TAA	4	1,365-2,900	1,536	ATG	TAA	3	1,370-2,905	1,536	ATG	TAA	5
tRNA^{Leu2}	L2	J	TAA	2,902-2,969	68	-	-	1	2,902-2,969	68	-	-	1	2,907-2,974	68	-	-	1
COII	-	J	-	2,969-3,646	678	ATT	T__	-1	2,969-3,647	679	ATT	T__	-1	2,974-3,653	680	ATT	TA_	-1
tRNA^{Lys}	K	J	TTT	3,647-3,721	75	-	-	-1	3,647-3,720	74	-	-	-1	3,654-3,727	74	-	-	0
tRNA^{Asp}	D	J	GTC	3,725-3,789	65	-	-	3	3,721-3,790	70	-	-	0	3,730-3,798	69	-	-	2
ATP8	-	J	-	3,790-3,945	156	ATC	TAA	0	3,790-3,945	156	ATA	TAA	-1	3,798-3,953	156	ATC	TAA	-1
ATP6	-	J	-	3,939-4,613	675	ATG	TAA	-7	3,939-4,610	672	ATG	TAG	-7	3,947-4,618	672	ATG	TAA	-7
COIII	-	J	-	4,597-5,385	789	ATG	TAA	-17	4,597-5,385	789	ATG	TAA	-14	4,605-5,393	789	ATG	TAA	-14
tRNA^{Gly}	G	J	TCC	5,388-5,454	67	-	-	2	5,388-5,454	67	-	-	2	5,396-5,462	67	-	-	2
ND3	-	J	-	5,454-5,802	352	GTG	TAA	-1	5,454-5,805	352	ATT	TAA	-1	5,462-5,815	354	ATA	TAA	-1
tRNA^{Ala}	A	J	TGC	5,808-5,870	63	-	-	0	5,808-5,871	64	-	-	0	5,816-5,876	61	-	-	0
tRNA^{Arg}	R	J	TCG	5,870-5,938	69	-	-	-1	5,871-5,937	67	-	-	-1	5,885-5,951	67	-	-	8
tRNA^{Asn}	N	J	GTT	5,935-6,002	68	-	-	-4	5,934-6,002	69	-	-	-4	5,949-6,016	68	-	-	-3
tRNA^{Ser1}	S1	J	TCT	6,001-6,069	69	-	-	-2	6,001-6,070	70	-	-	-2	6,012-6,084	73	-	-	-5
tRNA^{Glu}	E	J	TTC	6,073-6,139	67	-	-	3	6,069-6,135	67	-	-	-2	6,088-6,135	66	-	-	3
tRNA^{Phe}	F	N	GAA	6,139-6,206	68	-	-	-1	6,138-6,206	69	-	-	2	6,151-6,218	68	-	-	-3
ND5	-	N	-	6,207-7,882	1,676	ATG	TA_	0	6,206-7,880	1,675	ATG	T__	-1	6,218-7,892	1,675	ATG	T__	-1
tRNA^{His}	H	N	GTG	7,883-7,945	63	-	-	0	7,880-7,946	67	-	-	-1	7,893-7,956	64	-	-	0
ND4	-	N	-	7,947-9,275	1,329	ATG	TAA	1	7,949-9,274	1,326	ATG	TAG	2	7,963-9,288	1,326	ATG	TAG	6
ND4L	-	N	-	9,269-9,562	294	ATA	TAA	-7	9,268-9,561	294	ATA	TAA	-7	9,282-9,575	294	ATA	TAA	-7
tRNA^{Thr}	T	J	TGT	9,544-9,619	76	-	-	-19	9,553-9,620	68	-	-	-9	9,568-9,630	63	-	-	-8
tRNA^{Pro}	P	N	TGG	9,619-9,683	65	-	-	-1	9,623-9,688	66	-	-	2	9,627-9,694	68	-	-	-4
ND6	-	J	-	9,685-10,185	501	ATT	TAA	1	9,690-10,187	498	ATT	TAA	1	9,695-10,195	501	ATT	TAA	0

CYTB	-	J	-	10,185-11,324	1,140	ATG	TAA	-1	10,187-11,325	1,139	ATG	TA_	-1	10,195-11,335	1,141	ATG	T__	-1
tRNA^{Ser2}	S2	J	TGA	11,329-11,397	69	-	-	4	11,326-11,396	71	-	-	0	11,336-11,404	69	-	-	0
ND1	-	N	-	11,396-12,340	945	ATG	TAA	-2	11,394-12,338	945	ATG	TAA	-3	11,405-12,349	945	ATG	-	0
tRNA^{Leu1}	L1	N	TAG	12,342-12,407	66	-	-	1	12,339-12,405	67	-	-	0	12,351-12,415	65	-	-	1
16s rRNA	-	N	-	12,409-13,638	1,230	-	-	1	12,406-13,630	1,225	-	-	0	12,417-13,644	1,228	-	-	1
tRNA^{Val}	-	N	TAC	13,639-13,714	74	-	-	0	13,631-13,698	68	-	-	0	13,645-13,716	72	-	-	0
12s rRNA	-	N	-	13,713-14,476	764	-	-	0	13,699-14,471	773	-	-	0	13,717-14,488	772	-	-	0
AT-rich region	-	-	-	14,447-15,209	733	-	-	0	14,472-15,208	737	-	-	0	14,489-15,340	852	-	-	0

Table S7. Nucleotide composition of the complete mitogenomes of *Cysteochila lineata*, *Neoplerochila* sp. and *Plerochila australis.* AT-skew = (A-T)/(A+T); CG-skew = (G-C)/(G+C).

	Cysteochila lineata									
**Gene/region**	**A%**	**C%**	**G%**	**T%**	**A+T%**	**G+C%**	**AT-skew**	**GC-skew**	**Size (bp)**	**% (size)**
**COI**	31.5	18.6	15.6	34.3	65.8	34.2	-0.04	-0.09	1536	10.1
**COII**	37.3	18.3	11.3	33.1	70.4	29.6	0.06	-0.24	679	4.5
**COIII**	36.1	15.3	14.3	34.2	70.3	29.6	0.03	-0.03	789	5.2
**CYTB**	33.3	18.1	13.2	35.4	68.7	31.3	-0.03	-0.16	1140	7.5
**ATP6**	39.9	15.3	8.3	36.6	76.5	23.6	0.04	-0.30	675	4.4
**ATP8**	47.4	12.2	8.3	32.1	79.5	20.5	0.19	-0.19	156	1.0
**ND1**	47.8	16.3	10.8	25.1	72.9	27.1	0.31	-0.20	945	6.2
**ND2**	40.1	12.3	10.2	37.4	77.5	22.5	0.03	-0.09	970	6.4
**ND3**	41.0	13.3	11.6	34.2	75.2	24.9	0.09	-0.07	354	2.3
**ND4**	50.9	15.0	10.3	23.8	74.7	25.3	0.36	-0.19	1329	8.7
**ND4L**	49.0	15.0	7.5	28.6	77.6	22.5	0.26	-0.33	294	1.9
**ND5**	50.8	13.8	10.9	24.5	75.3	24.7	0.35	-0.12	1676	11.0
**ND6**	40.5	12.6	9.4	37.5	78.0	22.0	0.04	-0.15	501	3.3
**16s rRNA**	43.0	13.6	8.1	35.3	78.3	21.7	0.10	-0.25	1230	8.1
**12s rRNA**	44.5	13.9	7.5	34.2	78.7	21.4	0.13	-0.30	764	5.0
**Total PCGs**	41.7	15.5	11.6	31.2	72.9	27.1	0.14	-0.14	11044	72.6
**Total tRNAs**	40.6	11.9	10.0	37.5	78.1	21.9	0.04	-0.09	1489	9.8
**Total rRNAs**	43.6	13.7	7.9	34.9	78.5	21.6	0.11	-0.27	1994	13.1
**AT-rich region**	39.3	11.1	10.7	38.9	78.2	21.8	0.01	-0.02	733	4.8
**Complete mtDNA**	41.7	14.8	10.9	32.6	74.3	25.7	0.12	-0.15	15209	100.0

	Plerochila australis									
**Gene/region**	**A%**	**C%**	**G%**	**T%**	**A+T%**	**G+C%**	**AT-skew**	**GC-skew**	**Size (bp)**	**% (size)**
**COI**	32.3	17.5	15.4	34.8	67.1	32.9	-0.04	-0.06	1536	10.1
**COII**	38.4	15.8	11.0	34.8	73.2	26.8	0.05	-0.18	679	4.5
**COIII**	36.0	15.7	14.8	33.5	69.5	30.5	0.04	-0.03	789	5.2
**CYTB**	33.6	16.3	13.9	36.1	69.7	30.2	-0.04	-0.08	1140	7.5
**ATP6**	41.8	14.4	9.4	34.4	76.2	23.8	0.10	-0.21	672	4.4
**ATP8**	48.7	10.3	9.6	31.4	80.1	19.9	0.22	-0.04	156	1.0
**ND1**	49.5	16.1	10.7	23.7	73.2	26.8	0.35	-0.20	945	6.2
**ND2**	43.2	10.6	8.5	37.7	80.9	19.1	0.07	-0.11	972	6.4
**ND3**	42.4	12.7	10.5	34.5	76.9	23.2	0.10	-0.09	354	2.3
**ND4**	51.0	13.7	9.9	25.4	76.4	23.6	0.34	-0.16	1326	8.7
**ND4L**	49.7	12.2	7.5	30.6	80.3	19.7	0.24	-0.24	294	1.9
**ND5**	51.2	12.7	10.3	25.8	77.0	23.0	0.33	-0.10	1675	11.0
**ND6**	38.0	14.3	9.4	38.4	76.4	23.7	-0.01	-0.21	498	3.3
**16s rRNA**	43.5	12.3	8.2	36.0	79.5	20.5	0.09	-0.20	1225	8.1
**12s rRNA**	42.2	12.7	8.8	36.3	78.5	21.5	0.08	-0.18	773	5.1
**Total PCGs**	41.7	15.5	11.6	31.2	72.9	27.1	0.14	-0.14	11036	72.6
**Total tRNAs**	39.7	11.8	10.2	38.3	78.0	22.0	0.02	-0.07	1485	9.8
**Total rRNAs**	43.0	12.5	8.4	36.1	79.1	20.9	0.09	-0.20	1998	13.1
**AT-rich region**	39.4	13.8	12.1	35.8	75.2	25.9	0.05	-0.07	737	4.8
**Complete mtDNA**	42.1	14.0	10.9	33.0	75.1	24.9	0.12	-0.12	15208	100.0

	***Neoplerochila* sp.**									
**Gene/region**	**A%**	**C%**	**G%**	**T%**	**A+T%**	**G+C%**	**AT-skew**	**GC-skew**	**Size (bp)**	**% (size)**
**COI**	31.8	18.0	15.6	34.6	66.4	33.6	-0.04	-0.07	1536	10.0

**COII**	36.5	17.9	11.5	34.1	70.6	29.4	0.03	-0.22	680	4.4
**COIII**	36.4	15.8	13.8	34.0	70.4	29.6	0.03	-0.07	789	5.1
**CYTB**	34.6	16.6	12.9	35.9	70.5	29.5	-0.02	-0.13	1141	7.4
**ATP6**	40.0	14.0	9.1	36.1	76.1	23.1	0.05	-0.21	672	4.4
**ATP8**	49.4	14.1	6.4	30.1	79.5	20.5	0.24	-0.38	156	1.0
**ND1**	50.7	15.4	9.7	24.1	74.8	25.1	0.36	-0.23	945	6.2
**ND2**	42.8	12.3	8.8	36.0	78.8	21.1	0.09	-0.17	972	6.3
**ND3**	42.4	13.0	8.8	35.9	78.3	21.8	0.08	-0.19	354	2.3
**ND4**	51.9	13.9	10.3	24.0	75.9	24.2	0.37	-0.15	1326	8.6
**ND4L**	52.0	12.6	6.1	29.3	81.3	18.7	0.28	-0.35	294	1.9
**ND5**	51.6	13.7	9.6	25.1	76.7	23.3	0.35	-0.18	1675	10.9
**ND6**	38.3	10.6	8.4	42.7	81.0	19.0	-0.05	-0.12	501	3.3
**16s rRNA**	43.6	13.0	7.6	35.8	79.4	20.6	0.10	-0.26	1228	8.0
**12s rRNA**	44.0	11.6	8.0	36.0	80.0	19.6	0.10	-0.18	772	5.0
**Total PCGs**	42.4	14.7	10.8	32.0	74.4	25.5	0.14	-0.15	11041	72.0
**Total tRNAs**	40.1	12.4	10.1	37.5	77.6	22.5	0.03	-0.10	1479	9.6
**Total rRNAs**	43.8	12.6	7.8	35.9	79.7	20.4	0.10	-0.24	2000	13.0
**AT-rich region**	38.3	12.9	10.2	38.6	76.9	23.1	0.00	-0.12	852	5.6
**Complete mtDNA**	42.3	14.3	10.4	33.1	75.4	24.7	0.12	-0.16	15340	100.0

Table S8. Codon usage in the complete mitogenomes of three olive lace bug species (Hemiptera: Tingidae). Amino acids are labelled according to the IUPAC-IUB single letter codes. N - total number of occurrences in all protein coding genes, RSCU - relative synonymous codon usage.

Cysteochila lineata							
**Amino acid**	**Codon**	**N**	**RSCU**	**Amino acid**	**Codon**	**N**	**RSCU**
F	UUU	171	1.36	Y	UAU	195	1.4
	UUC	80	0.64		UAC	84	0.6
L	UUA	356	3.15	H	CAU	78	1.17
	UUG	71	0.63		CAC	55	0.83
	CUU	81	0.72	Q	CAA	138	1.44
	CUC	37	0.33		CAG	54	0.56
	CUA	103	0.91	N	AAU	271	1.44
	CUG	30	0.27		AAC	105	0.56
I	AUU	219	1.58	K	AAA	413	1.6
	AUC	59	0.42		AAG	103	0.4
M	AUA	287	1.71	D	GAU	62	1.48
	AUG	49	0.29		GAC	22	0.52
V	GUU	46	1.12	E	GAA	108	1.49
	GUC	21	0.51		GAG	37	0.51
	GUA	77	1.88	C	UGU	41	1.55
	GUG	20	0.49		UGC	12	0.45
S	UCU	78	1.57	W	UGA	70	1.54
	UCC	42	0.84		UGG	21	0.46
	UCA	89	1.79	R	CGU	17	1.19
	UCG	21	0.42		CGC	9	0.63
P	CCU	45	0.99		CGA	18	1.26
	CCC	50	1.1		CGG	13	0.91
	CCA	70	1.55	S	AGU	47	0.94
	CCG	16	0.35		AGC	25	0.5
T	ACU	81	1.24		AGA	79	1.59
	ACC	67	1.03		AGG	17	0.34
	ACA	97	1.49	G	GGU	36	1.35
	ACG	16	0.25		GGC	9	0.34
A	GCU	23	1.42		GGA	26	0.97
	GCC	14	0.86		GGG	36	1.35
	GCA	24	1.48				
	GCG	4	0.25				

Plerochila australis							
**Amino acid**	**Codon**	**N**	**RSCU**	**Amino acid**	**Codon**	**N**	**RSCU**
F	UUU	193	1.47	Y	UAU	219	1.4
	UUC	69	0.53		UAC	93	0.6

L	UUA	324	3.19	H	CAU	78	1.28
	UUG	67	0.66		CAC	44	0.72
	CUU	80	0.79	Q	CAA	116	1.49
	CUC	25	0.25		CAG	40	0.51
	CUA	92	0.9	N	AAU	263	1.43
	CUG	22	0.22		AAC	105	0.57
I	AUU	241	1.55	K	AAA	431	1.64
	AUC	69	0.45		AAG	96	0.36
M	AUA	299	1.71	D	GAU	65	1.55
	AUG	51	0.29		GAC	19	0.45
V	GUU	63	1.55	E	GAA	108	1.54
	GUC	16	0.39		GAG	32	0.46
	GUA	64	1.57	C	UGU	53	1.43
	GUG	20	0.49		UGC	21	0.57
S	UCU	60	1.19	W	UGA	69	1.59
	UCC	35	0.69		UGG	18	0.41
	UCA	97	1.92	R	CGU	8	0.73
	UCG	18	0.36		CGC	9	0.82
P	CCU	56	1.27		CGA	17	1.55
	CCC	43	0.98		CGG	10	0.91
	CCA	62	1.41	S	AGU	54	1.07
	CCG	15	0.34		AGC	27	0.53
T	ACU	76	1.17		AGA	82	1.62
	ACC	75	1.16		AGG	32	0.63
	ACA	92	1.42	G	GGU	29	1.05
	ACG	16	0.25		GGC	16	0.58
A	GCU	20	1.04		GGA	26	0.95
	GCC	17	0.88		GGG	39	1.42
	GCA	35	1.82				
	GCG	5	0.26				

***Neoplerochila* sp.**							
**Amino acid**	**Codon**	**N**	**RSCU**	**Amino acid**	**Codon**	**N**	**RSCU**
F	UUU	223	1.53	Y	UAU	214	1.46
	UUC	69	0.47		UAC	80	0.54
L	UUA	302	3.06	H	CAU	68	1.35
	UUG	51	0.52		CAC	33	0.65
	CUU	96	0.97	Q	CAA	122	1.53
	CUC	32	0.32		CAG	37	0.47
	CUA	86	0.87	N	AAU	309	1.37
	CUG	26	0.26		AAC	143	0.63
I	AUU	290	1.54	K	AAA	407	1.56
	AUC	86	0.46		AAG	116	0.44
M	AUA	319	1.73	D	GAU	53	1.38
	AUG	49	0.27		GAC	24	0.62

V	GUU	55	1.53	E	GAA	102	1.61
	GUC	9	0.25		GAG	25	0.39
	GUA	68	1.89	C	UGU	49	1.48
	GUG	12	0.33		UGC	17	0.52
S	UCU	62	1.07	W	UGA	60	1.35
	UCC	38	0.65		UGG	29	0.65
	UCA	101	1.74	R	CGU	15	1.07
	UCG	13	0.22		CGC	7	0.5
P	CCU	65	1.43		CGA	22	1.57
	CCC	44	0.97		CGG	12	0.86
	CCA	60	1.32	S	AGU	58	1
	CCG	13	0.29		AGC	34	0.58
T	ACU	73	1.05		AGA	109	1.88
	ACC	81	1.17		AGG	50	0.86
	ACA	104	1.5	G	GGU	24	1.1
	ACG	19	0.27		GGC	10	0.46
A	GCU	12	0.84		GGA	24	1.1
	GCC	12	0.84		GGG	29	1.33
	GCA	29	2.04				
	GCG	4	0.28				
